# Supplementary material for: Maternal and Fetal Outcomes after Prior Mid-Trimester Uterine Rupture: A Systematic Review with Our Experience
Source: Medicina (Kaunas). 2021 Nov 24;57(12):1294. doi: 10.3390/medicina57121294 (PMC8704285; doi:10.3390/medicina57121294)
Supplement: Supplementary file 1 [file medicina-57-01294-s001.zip › Suppelemetal Files.pdf]

Supplemental File S1. The search strategy.

PubMed

#1 “Uterine rupture”[TIAB] OR Uterine rupture [MeSH] 6250

#2 Pregnancy Trimester, Second [MeSH] OR “second trimester” OR “midtrimester” OR “mid-trimester” OR “13 weeks” OR “14 weeks” OR “15 weeks” OR “16 weeks” OR “17 weeks” OR “18 weeks” OR “19 weeks” OR “20 weeks” OR “21 weeks” OR “22 weeks” OR “23 weeks” OR “24 weeks” OR “25 weeks” OR “26 weeks” OR “27 weeks” OR “28 weeks” 148,704

#3 #1 AND #2 608

#4 “subsequent pregnancy” OR “next pregnancy” OR “consequent pregnancy” OR “successive pregnancy” OR “later pregnancy” 3849

#5 #3 AND #4 24

#6 “after uterine rupture” OR “prior uterine rupture” OR “previous uterine rupture” 17

#7 #5 OR #6 40

Cochrane (Trials)

#1 “Uterine rupture”[TIAB] 163

#2 Uterine rupture [MeSH] 39

#3 #1 OR #2 178

#4 Pregnancy Trimester, Second [MeSH] 696

#5 “second trimester” OR “midtrimester” OR “mid-trimester” OR “13 weeks” OR “14 weeks” OR “15 weeks” OR “16 weeks” OR “17 weeks” OR “18 weeks” OR “19 weeks” OR “20 weeks” OR “21 weeks” OR “22 weeks” OR “23 weeks” OR “24 weeks” OR “25 weeks” OR “26 weeks” OR “27 weeks” OR “28 weeks” 55,517

#6 #4 OR #5 52,706

#7 #3 AND #6 21

#8 “subsequent pregnancy” OR “next pregnancy” OR “consequent pregnancy” OR “successive pregnancy” OR “later pregnancy” 265

#9 #7 AND #8 0

#10 “after uterine rupture” OR “prior uterine rupture” OR “previous uterine rupture” 2

#11 #9 OR #10 2

Scopus

#1 TITLE-ABS-KEY ("uterine rupture") 5,819

#2 TITLE-ABS-KEY (“second trimester” OR “midtrimester” OR “mid-trimeseter” OR “13 weeks” OR “14 weeks” OR “15 weeks” OR “16 weeks” OR “17 weeks” OR “18 weeks” OR “19 weeks” OR “20 weeks” OR “21 weeks” OR “22 weeks” OR “23 weeks” OR “24 weeks” OR “25 weeks” OR “26 weeks” OR “27 weeks” OR “28 weeks”) 197,618

#3 #1 AND #2 604

#4 TITLE-ABS-KEY (“subsequent pregnancy” OR “next pregnancy” OR “consequent pregnancy” OR “successive pregnancy” OR “later pregnancy”) 18,511

#5 #3 AND #4 32

#6 TITLE-ABS-KEY (“after uterine rupture” OR “prior uterine rupture” OR “previous uterine rupture”) 57

#7 #5 OR #6 88

Supplemental File S2. PICOS criteria for inclusion of systematic review.

**Population**	Pregnant women
**Intervention**	Prior uterine rupture at mid-trimester
**Comparison**	No prior uterine rupture
**Outcome**	Obstetric outcome (the rates of PAS, hysterectomy, PPH)<br>Maternal outcome (the rate of transfusion, death)
**Study design**	Case report, retrospective or prospective cohort studies, case-control study, and randomized controlled trials

Abbreviations: PICOS, Patient/Population, Intervention, Comparator, Outcome, Study; PPH, postpartum hemorrhage; PAS, placenta accreta spectrum.
